# Current News and Opinion

**Published:** 1883-03

**Authors:** 


### Current News and Opinion.

The New York State Medical Society held its twenty-seventh annual meeting, and there was no "great fight," as had been expected; on the contrary the utmost harmony prevailed, and the result of the Code business was a foregone conclusion to the knowing ones.

Dr. E. R. Squibb offered the following:

Whereas, The Special Committee on the Code of Ethics, in its report at the last annual meeting, recommended a change in one part of the Code which was more in the nature of a revolution than of a revision, and therefore may be more radical than was expected or desired by the constituency of this society ; and

*Whereas*, That report was adopted at a session wherein only fifty-two members voted in the affirmative, and thus legislated for the entire profession of the State on a subject of vital importance in a direction which may not have been anticipated or desired by the profession at large; therefore,

*Be it resolved*, That all the action taken at the annual meeting of 1881, in regard to changing the Code of Ethics, be repealed, leaving the Code to stand as it was before such action was taken.

*Resolved*, That a new Special Committee of five be nominated by the Nominating Committee of the Society, and be appointed by the Society to review the Code of Ethics, and to report at the annual meeting of 1884 any changes in the Code that may be deemed advisable.

*Resolved*, That the report of this Committee be discussed at the meeting of 1884, and be then laid over for final action at the meeting of 1885.

It was voted that it be the special order for the evening session. After three hours' discussion in committee of the whole, the question was called and the ayes and nays being taken, the vote stood: Ayes, 99 ; nays, 105.

So the new Code is the Code.

Dr. Roosa's resolution, introduced directly the above result was announced, read, in spirit, that the only discipline that could be exercised over a physician by his brethren was in regard to acts unworthy of a gentlemen and a physician.

The action on Dr. Roosa's resolution was immediately postponed a twelfthmonth.

The meeting was well nigh a "specialists'" meeting, diseases of the eye and ear being by far the most prominent topic, concerning which papers were prepared and read.

This made it but moderately interesting, at least for the general practitioner, whereas, to our minds, it is he who should see that a one-sided meeting does not again occur, by himself preparing papers on subjects of wider interest—hygiene, preventive medicine, etc.

The Cartwright Lectures of the Alumni Association of the College of Physicians and Surgeons for the present year were delivered at the Hall of the Young Men's Christian Association, corner of Fourth Avenue and Twenty-third Street, by Dr. W. T. Belfield, of Chicago, on the evenings of February 19th, 21st, 24th and 27th at eight o'clock. Subject: "The Relations of Micro-organisms to Disease."

The Annual Commencement of the Medical and Dental Departments of the University of Maryland, will be held in conjunction, at the Academy of Music March 15, 1883. Physicians and Dental Specialists are invited to be present.

Congress has struck from the Tariff Bill the item placing a duty of 10 per cent. on the sulphate and salts of quinia and cinchonidia.

New Remedy in Diphtheria.—A German apothecary, R. Münch (Kronen-Apotheke in Leipsic-Sohlis), who enjoys a great reputation for veracity and reliability amongst those who know him, recommends in No. 27 of the Pharm. Centr. Anzeiger, "as a new remedy in diphtheria, and the effect" of which he had noticed on his own seven-year-old daughter—oleum terebinthinæ rectificatum. Children take one teaspoonful morning and night; adults, a tablespoonful. In children tepid milk is given after it; it might also be mixed with the same. The effect of this remedy, which has of late been highly praised by different authors, is said to be really a miraculous one. Within already half an hour after the administration of the drug, a bright redness begins to spread from the margin of the diphtheritic exudation, and this redness becomes generally diffused over and taking the place of the false membrane, and the disease is said to disappear within twenty-four hours without leaving the slightest trace. While this wonderful effect is said to be invariably met with when the remedy is made use of at the very commencement of the disease, those who recommend it so highly contend that it is also successful, only less rapidly, in cases that have already progressed for several days.

The Mississippi Valley Association at Cincinnati March 7, 8 and 9th. Subjects for discussion:

1. Nervous and muscular affections dependent on dental irritation.
2. Etiology and pathology of dental caries; treatment by filling.
3. Periodentitis and alveolar abscess; pathology and treatment.
4. Prosthetic dentistry; restoration of features and expression—how best accomplished.
5. Fitting artificial crowns to roots of natural teeth—new and old methods.
6. Reports of case in practice.

Intestinal Obstruction Relieved by Massage.—Dr. Bitterlin reports a case of intestinal occlusion accompanied with much pain, vomiting of fecaloid matter, hiccough continuing in spite of treatment for eight days, finally relieved by kneading and malaxation of the belly. The manipulation was very painful. Some instants after, violent colic came on and gurglings, the bowels shortly afterwards moved and the patient recovered. Dr. Bitterlin mentions a second case in which he was called in consultation, where the same treatment was followed by the same happy results.—*L'Union Medical.*

ASPIRATION OF A SEROUS FLUID IN THE PERICARDIUM. RECOVERY.—A boy twelve years of age suffered first from acute rheumatism. After two weeks, pericarditis set in, and suddenly the patient became asphyctic, the veins of the thorax being overfilled, and the action of the heart very irregular. An anspirator needle was inserted three cm. from the sternum, into the second intercostal space, and then, no effect resulting, into the third intercostal space. One hundred grms. of a serous fluid were aspirated, and the patient recovered quickly.—*Correspondenz Blatt. f. Schweizer Aertze.*

DR. W. ST. GEORGE ELLIOTT, the well-known American dentist of London, is delivering a course of lectures upon operative dentistry, at the National Dental College, Great Portland Street, London, England.

THE Alabama Dental Association will hold its Fourth Annual Meeting in Montgomery on the second Tuesday in April. The State Board of Dental Examiners will meet at the same time and place.

DR. B. W. RICHARDSON of London declared, in a recent address, that he had never seen a healthy child, nor one that had not in it either some ancestral or latent constitutional disease.

IT IS STATED on good authority that the *British Medical Journal* has been advertising a work entitled "Diseases of the Prostate in Both Sexes," by David Jones, M.D.

CHAUVEAU announces that he has found the microbe of puerperal fever. It were desirable that vaccination with it in an attenuated form may prove practicable.

THE Forty-second Annual Commencement of the University of the City of New York, Medical Department, will be held at the Academy of Music March 13, 1883.

BODY SNATCHING is quite prevalent, and the daily press has a rich mine of horrors wherewith to work on the easily excitable morbid fancies of their readers.

THE Vermont Dental Sooiety will hold its Seventh Annual Meeting in the Bates House, Rutland, Vt., on March 21, 22 and 23, commencing at 7:30 P.M.

MULOT, in *Le Progres Medical*, reports a case of primary cancer of the gall bladder which is an extremely rare pathological condition.

"SYMPOSIA" seem to be the rule with the *N. Y. Medical Journal.* After carbolic acid in January, came kidney and stone in February.

THE Brooklyn Dental Society will hold its next meeting on the 12th inst. at the residence of Dr. Van Wart, Noble Street, Greenpoint.

THE New York College of Dentistry will hold its Seventeenth Annual Commencement on the 6th of March, at Chickering Hall.

OUTSIDE New York State they speak of it as "*the recent disaster at Albany.*"

